# A database of chlorophyll and water chemistry in freshwater lakes

**DOI:** 10.1038/s41597-020-00648-2

**Published:** 2020-09-22

**Authors:** Alessandro Filazzola, Octavia Mahdiyan, Arnab Shuvo, Carolyn Ewins, Luke Moslenko, Tanzil Sadid, Kevin Blagrave, Mohammad Arshad Imrit, Derek K. Gray, Roberto Quinlan, Catherine M. O’Reilly, Sapna Sharma

**Affiliations:** 1 Department of Biology, York University, Toronto, Canada; 2 Department of Biology, Wilfrid Laurier University, Waterloo, Canada; 3 Department of Geography-Geology, Illinois State University, Illinois, USA

**Keywords:** Freshwater ecology

## Abstract

Measures of chlorophyll represent the algal biomass in freshwater lakes that is often used by managers as a proxy for water quality and lake productivity. However, chlorophyll concentrations in lakes are dependent on many interacting factors, including nutrient inputs, mixing regime, lake depth, climate, and anthropogenic activities within the watershed. Therefore, integrating a broad scale dataset of lake physical, chemical, and biological characteristics can help elucidate the response of freshwater ecosystems to global change. We synthesized a database of measured chlorophyll a (chla) values, associated water chemistry variables, and lake morphometric characteristics for 11,959 freshwater lakes distributed across 72 countries. Data were collected based on a systematic review examining 3322 published manuscripts that measured lake chla, and we supplemented these data with online repositories such as The Knowledge Network for Biocomplexity, Dryad, and Pangaea. This publicly available database can be used to improve our understanding of how chlorophyll levels respond to global environmental change and provide baseline comparisons for environmental managers responsible for maintaining water quality in lakes.

## Abstract (summary)

**Table:** 

Measurement(s)	chlorophyll a • phosphorus atom • nitrogen atom • dissolved carbon atom in water • dissolved oxygen in water • volume • lake surface area • depth of water • pH
Technology Type(s)	digital curation
Factor Type(s)	geographic location
Sample Characteristic - Environment	freshwater lake
Sample Characteristic - Location	Earth (planet)

Machine-accessible metadata file describing the reported data: 10.6084/m9.figshare.12928352

## Background & Summary

Lake water accounts for less than 1% of the world’s surface freshwater supply, but provides critical ecosystem services, including consumption, transportation, agriculture, and recreation, in addition to habitat for over 100,000 species of invertebrates, insects, animals, and plants^1–3^. However, freshwater lakes are vulnerable to the effects of water fouling, nutrient enrichment, and alterations in climate and land use owing to their sensitivity to local and global environmental changes^4–6^. Alterations in biological and chemical lake processes can affect how and when freshwater resources can be used. Particularly, increases in lake chlorophyll levels can impact water quality through alterations in colour and odor^7^, dissolved oxygen availability^8^, and overall lake production^9^.

Chlorophyll *a* (chla) is frequently used as a straightforward and suitable representative measurement of lake productivity and water quality^10–12^. Many environmental assessments commonly use chla as a biological indicator for determining lake trophic status^10,13^. In freshwater ecology, chla also functions as a good proxy for other biological variables, such as primary production, and is often included as a covariate in limnological studies^14^. Chla is therefore routinely measured in water quality programs across the globe, making a good candidate for the focus of a water quality database with broad spatial coverage.

To discern the limnological processes that determine chla in lakes requires consideration of water chemistry, lake morphometry, and the landscape setting. While lakes naturally vary in their chla concentrations owing to seasonal fluctuations and climate variability, they can also respond to anthropogenic influences such as nutrient inputs^15^. Anthropogenic sources of nutrient loadings in lakes include runoff from the surrounding watershed from land use changes^16,17^, atmospheric deposition^18^, and sewage discharge^19^. Furthermore, individual lake properties such as surface area, depth, and volume can mediate the temperature, productivity, and energy flow of a lake^20^. Accordingly, water chemistry (defined here as total phosphorus, total nitrogen, dissolved organic carbon, and dissolved oxygen) as well as numerous morphometric characteristics were included in the assembly of this database.

There are two main methods for generating chla data, either from model-inferred estimates using remotely sensed images or through *in situ* sampling. There are chla levels inferred from remote sensing^21,22^ that can be effective for comparisons among lakes, but these are less common because there is significant error surrounding the separation of turbidity from light attenuation in the water column^23,24^. Similarly, the extent of *in situ* measurements can be restricted because certain lakes are difficult to access (e.g. high alpine, or arctic). Ideally, a chla database would have both modelled and field measurements to allow users the option to trade-off spatial coverage for accuracy.

Building on recent extensive national water quality databases^25^, we fill a strong need for a cohesive and broad-scale database of water quality worldwide. The incentive to assemble this database of lake chla, water chemistry, and morphometric characteristics was to identify chla patterns over broad spatial and temporal scales. Other applications of this database include and are not limited to identifying which environmental stressors (e.g. climate, nutrient or anthropogenic factors) are most important in driving changes in water quality, specifically chla. Using the published scientific literature and online data repositories, we conducted a systematic review to acquire instances where chla has been measured. Here, we present a database of wide spatial coverage of chla from 11,959 lakes distributed across 72 countries collected *in situ* or by satellites. From these same data sources, we also acquired information about lake morphometry and water chemistry as they are highly correlated with chla concentrations. We provide a summary of these data and associated variables to serve as a tool in ecological research and freshwater management.

## Methods

### Data acquisition

**Fig. 1:**
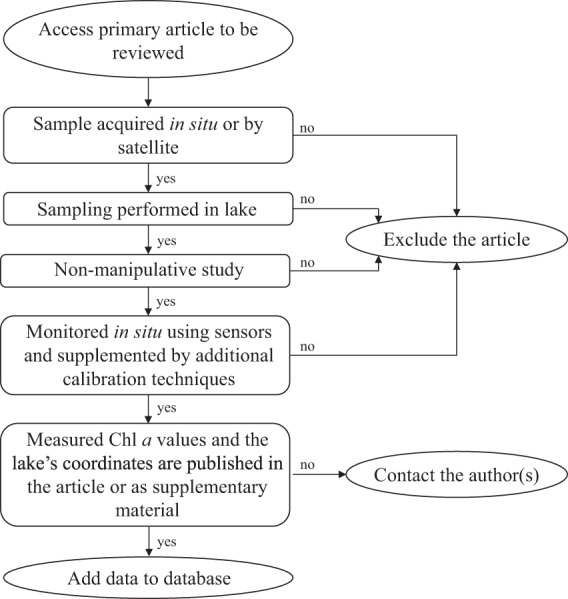
Workflow for all datasets included in the chlorophyll and water chemistry database.

We obtained data by conducting a systematic review of the literature and searching for published repositories in online databases. We first conducted a systematic review to identify relevant primary articles using “chlorophyll” and “lake*” as citation search terms in Web of Science between the years 2000 and 2018. From these published manuscripts, we acquired chla and other water chemistry data for 11,959 lakes worldwide. Papers that were not primary articles or were not in a field relating to limnology were excluded. We screened 3322 articles published between 2000 and 2018 because this timeframe represents more recent lake conditions (e.g. post zebra mussel invasion in North America) with minimal repetition. We excluded articles if the methods used to collect water quality data violated the following criteria: i) were not sampled in the lake (i.e. from a sediment core); ii) were collected in a manipulative study (i.e. from a mesocosm or other experimental modification of the lake’s water chemistry); or iii) were monitored *in situ* using sensors that were not supplemented by additional calibration techniques^26^ (Fig. 1). All lakes also required reporting of latitude and longitude. If an article did not violate any criteria, we extracted data from tables, in text, or through the digitization of figures using WebPlotDigitizer (https://apps.automeris.io/wpd/) either from the article or from the supplementary data (Fig. 1). Extracted data included values for chla, total phosphorus, total nitrogen, dissolved organic carbon, and dissolved oxygen. If data were unavailable from the manuscript, we contacted the study authors to request their data (Fig. 1). We also collected data on lake volume, surface area, mean depth, maximum depth, Secchi depth, and pH when available within the study.

We found an additional 15 online data repositories that contained lake chla measurements and other water chemistry data for 10,997 lakes using the online search engines Dryad (https://datadryad.org/), The Knowledge Network for Biocomplexity (KnB - https://knb.ecoinformatics.org/), Github (https://github.com/) and Google Dataset Search (https://toolbox.google.com/datasetsearch) also using the search terms “chlorophyll” and “lake*”. Information on each of the repositories can be found in Table 1. The data repositories were predominantly from the USA. The repository with the greatest number of observations and lakes was, by far, repo14 (92% of all observations and 69% of all lakes) that was a previous data compilation effort by Soranno *et al*.^25^ across multiple government agencies and research partners (Table 1). The methods varied for observations within this database but are well documented by the authors^25^. The sources of data obtained from these repositories were diverse, coming from government programs, independent research groups, Long-Term Ecological Research sites, and non-profit monitoring agencies.

**Table 1:** Information about each of the data repositories that were obtained online including the number of lakes, number of observations, timeframe of surveys, and a relevant study that utilized the data.

ID	Name	Lakes	Observations	Time frame	Relevant study	Notes
Repo1	Ecology under lake ice	39	1231	1969–2017	Hampton *et al*. (2016) Ecology Letters^39^	Paired winter and summer observations
Repo2	Limnological data and depth profile from Oneida Lake	1	222	1975–2018	Karatayev *et al*. (2014) PLoS One^40^	Measured weekly and averaged from five different locations
Repo3	Transparency, geomorphology, and mixing regime explain variability in trends in lake temperature and stratification across northeastern North America	215	219	1975–1985	Richardson *et al*. (2017) Water^41^	Samples were measured in 1975, 1985, or both.
Repo4	The European Multi Lake Survey (EMLS) dataset of physical, chemical, algal pigments and cyanotoxin parameters 2015	332	345	2015	Mantzouki *et al*. (2018) Nature Scientific Data^42^	Surveyed in the summer. Some sampling points were reclassified as within the same lake
Repo5	Water quality database	2,168	9,568	1967–2019		An online database that joins data collected from multiple US government Agencies
Repo6	The Lake Inventory Program (formerly known as the Lake Survey Program)	96	103	1974–2010		Samples were taken during the summer months at varying sampling depths and averaged
Repo7	National Aquatic Resource Surveys	1,059	1,162	2007	Pollard *et al*. (2018) Bulletin Limnology and Oceanography^43^	An integrated sampler was used to collect chla data at the centre of the lake
Repo8	McMurdo Dry Valleys Chlorophyll-A Concentrations in Lakes	7	102	1993–2016	Burnett *et al*. (2006) Arctic, Antarctic, and Alpine Research^44^	Sampling is conducted below permanent ice-cover in summer months
Repo9	Lake Kasumigaura Database	1	476	1977–2016	Takamura & Nakagawa (2012) Ecological Research^45^	Twelve stations within the lake are sampled monthly
Repo10	Cascade Project at North Temperate Lakes LTER High Frequency Sonde Data from Food Web Resilience Experiment 2008–2011	2	8	2008–2011	Gries *et al*. (2016) Ecological Informatics^46^	Samples were collected at 5-minute intervals during the summer and averaged for the year
Repo11	Lake Metabolism at North Temperate Lakes LTER 2000	24	24	2000	Gries *et al*. (2016) Ecological Informatics^46^	Measurements were taken in July and August
Repo12	Landscape Position Project at North Temperate Lakes LTER: Chlorophyll 1998–2000	49	52	1998–2000	Gries *et al*. (2016) Ecological Informatics^46^	Samples were taken two times or monthly in the summer at three depths.
Repo13	Unpublished data, Massachusetts Department of Environment Protection, lake water chemistry data, 1995–2004	111	111	1999–2004		Five sampling events interspersed throughout the summer and averaged
Repo14	LAGOS-NE: a multi-scaled geospatial and temporal database of lake ecological context and water quality for thousands of US lakes	8,218	209,732	1933–2013	Soranno *et al*. (2017) GigaScience^25^	A dataset compilation across government agencies and universities in the USA

### Chlorophyll data

**Fig. 2:**
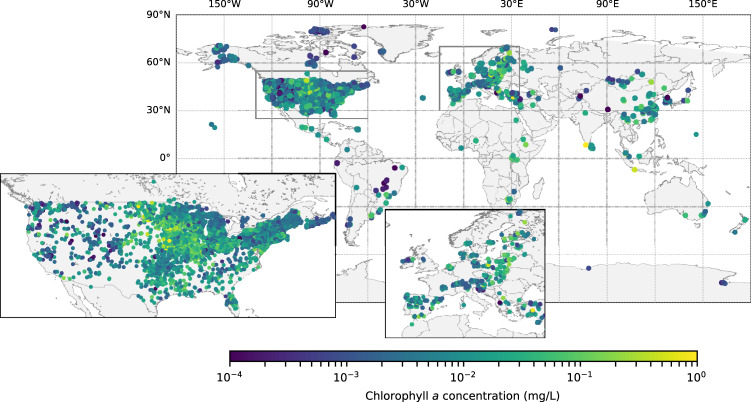
The distribution of lakes included in database that have measured chlorophyll values. Insets are provided for the USA and Europe to better separate the high density of observations from lakes in these areas.

Our team acquired chla data for 228,168 unique survey instances in 11,959 lakes distributed across 72 countries and on every continent including Antarctica (Fig. 2). In all but 47 instances, data were measured *in situ*. In 10 datasets, chlorophyll was estimated using remote sensed data from satellite imagery. Although remote sensed data can be less precise than *in situ* surveys, we included this data because it provides estimates in lakes that are difficult to access. There were 37 instances where it was unclear which methodologies were used that are identified in the *methodsData.csv*. Each chla measurement was converted to standardized units (mg L^−1^) and corresponds with the lake’s latitude, longitude, and the year in which the measurement was taken (Table 2). In some cases, the same lake was sampled in multiple locations (which were associated with different coordinates within the lake’s perimeter) and/or sampled multiple times within the same year (e.g. monthly; which was associated with a sampling date and not just the year). Almost all datasets used surface measurements (41.4%) or an integrated water sample (23.8%). Only 7.7% of collected datasets used a specific depth for their measurements (the remaining were undescribed). The deepest sample collected was 250 m below the surface from Lake Baikal^27^. The detection limits for studies were often 0.1 μg L^−1^ or lower (71% of collected datasets), although some were coarse including 6% of collected datasets that had detection limits at 100 μg L^−1^. We flagged observations where the detection limits were greater than the observed value of chla (<1.6% of observations) because these values may be inaccurate and should be treated with caution. For instance, values of zero are likely not true zeros but may represent chla measurements below the detectable limits of the method used. There were 454 observations (0.12%) that had zero values.

**Table 2:** Table attributes and descriptions from database of chlorophyll values in freshwater lakes (ChlData.csv).

Attribute (column header)	Description of attribute	Data with values (%)
***ChlaData.csv***		
uniqueID	Unique identifier for each respective survey instance that exists across all datasets within this database	
UniqueLakeName	Unique lake identifier for reference across studies.	
StudyID	Study identifier to be connected to the data	
Year	Year that lake was surveyed. Can be discrete (e.g. 2005, 2006) or a range of years where the values were averaged (e.g. 2005–2007).	
Month	Month that lake was surveyed as a number	
Lat	Latitude of survey instance (decimal degrees)	
Lon	Longitude of survey instance (decimal degrees)	
LakeName	Name of lake as identified within the manuscript or data repository	
ChlaValues	Average concentration of chlorophyll a in freshwater lakes at each survey instance (mg L^−1^)	100
TP	Average concentration of total phosphorus in freshwater lakes at each survey instance (mg L^−1^)	49.0
TN	Average concentration of total nitrogen in freshwater lakes at each survey instance (mg L^−1^)	17.3
DOC	Average concentration of dissolved organic carbon in freshwater lakes at each survey instance (mg L^−1^)	4.6
DO	Average concentration of dissolved oxygen are in freshwater lakes at each survey instance (mg L^−1^)	< 1
LakeVolume	The volume of the lake that was surveyed in m^3^	< 1
SurfaceArea	The measured surface area of the lake that was surveyed (km^2^)	92.9
Depth.mean	The average depth of the lake that was sampled in meters	31.9
Depth.max	The maximum depth of the lake that was sampled in meters	82.5
Secchi	The distance underwater that the Secchi depth was no longer visible from the surface (meters)	85.8
pH	The pH of sampled water	5.7
Chla.flag	An identifier to highlight Chla values that are below the detection limits listed in the study and thus subjected to inaccuracies.	1.5

Sampling method techniques varied including analysis by spectrophotometry, fluorometry, or the methods by Eaton and Franson^28^. A comprehensive discussion of the details of each of the standard methods of chla extraction can typically be found in individual manuscripts. Generally, water samples were passed through a filter, and then chla was extracted from the organism atop the filter using an organic solvent (e.g. acetone or ethanol). The chla concentrations were then determined by a spectrophotometer (to record light absorbance of chla at a specific wavelength) or by a fluorometer (to record light fluorescence of chla at a specific wavelength). Other methods of data collection included high performance liquid chromatography and sonication/freeze-thaw method.

The chlorophyll values reported were often aggregated values that were collected across multiple profiles of the water column, different points within the same lake (spatial), or the same location but over multiple times (temporal). We documented the replication within each of the collected datasets when provided for each of these three categories (column, spatial, and temporal) in the *methodsData.csv* (Table 3). We included both a qualitative and quantitative description of replication in these three categories from each individual dataset. The measurement type would either be described as a *raw* value, meaning it was collected and reported from a single observation, or it would be reported as an aggregate statistic (i.e. mean or median). The *NumObs* column within the *methodsData.csv* represents the number of values that were extracted from the respective study or online repository. This number was typically smaller than the *Replicate* column which represented the number of observations collected by the original data contributors of the study or repository. The *Replicate* column can often be estimated by multiplying the number of replicates in the column profile, the areas sampled within the lake (spatial), and the number of times it was sampled (temporal). However, there were cases where the *Replicate* column was not divisible by these three categories because of uneven sampling. For example, one lake may have been sampled at three different depths but another sampled at only one. When multiple depths were provided, we calculated an average for each water chemistry variables to create an integrated water sample and provide the details of the depths surveyed in the *methodsData.csv*. In all other cases (i.e. temporal and spatial), we maintained each replicate within a lake as a separate observation in our dataset when the authors provided this information. There was considerable variability in the number of replicates that were collected between studies and repositories. For instance, repo10 collected values every five minutes (n = 254,527) that were collated into annual averages over four years for two lakes (n = 8). By contrast other lakes were sampled considerably less, such as a series of Patagonian and Pampean lakes that were sampled once annually for two years^29^. We provide details of all available data on replication in the *methodsData.csv* file to allow for accurate comparisons between studies.

**Table 3:** Table attributes and descriptions for meta-data files on studies (MS.citations.csv), data repositories (Repo.citations.csv), and methods of data collection (methodsData.csv).

Data label	Description
***MS.citations***	
StudyID	Identifier for the published study
Title	Title of published study
Authors	Authors of published study
Source Title	Journal that the study was published in
Publication Year	Year that the study was published
Volume	Volume from journal
Issue	Issue from journal
Beginning Page	First page in the journal that the study was published
Ending Page	Last page in the journal that the study was published
DOI	Digital Object Identifier associated with study
Total Citations	Total number of citations associated with the study as of October 2018
Exclude	Whether the study was excluded from the database
reason.simplified	A simplified reason why the study was not used
***Repo.citations.csv***	
StudyID	Study identifier to be connected to the data
StudyName	Name of study
Link	Link where data were obtained from
Author	Authors that were listed in study
Title	Title of study
DataSource	Source data were acquired from including databases, repositories, or online searches
Year	Year the dataset was published
Included	Whether the dataset was processed and added to the main dataset
***methodsData.csv***	
StudyID	Study identifier to be connected to the data
Year	Year that the study was published
Chl method	The method of which the chlorophyll sample was measured
MeasurementType	The type of value as either the mean, median, or raw (unaggregated)
DetectionLimits	The lowest recorded measurement within the study
Survey.Type	The collect method, either *in situ* or from satellite/modelling.
Depth.qual	A qualitative description of the depth that the measurement was taken such as surface, integrated or specific depth.
Depth.quant	A description of the depths that the measurement was collected
Column.rep	The number of depths that an integrated measurement was collected
Replicate	The total number of measurements that were included in generating the mean or number of observations. Includes replicates in column, area of lake, and time.
Spatial.rep	The number of locations within or among lakes that samples were collected
Spatial.qual	A description of the locations within a lake that a sample was collected (e.g. integrated, center, shoreline).
Temporal.rep	The number of measurements over time that were collected
Temporal.qual	A description of the time interval that was used for sampling.
StartDay	The day of the month the surveys began
StartMonth	The month of the year that the surveys began
StartYear	The year that the surveys began
EndDay	The day of the month the surveys ended
EndMonth	The month of the year that the surveys ended
EndYear	The year that the surveys ended
DepthDetails	A description of the sampling that was conducted on the column
DepthShallow	The shallowest depth that a sample was collected
DepthMean	The average depth samples were collected
DepthDeep	The maximum depth that a sample was collected. -999 represents the bottom of the lake.
NumObs	The total number of observations that are present in the study that are included in the database.

### Water chemistry and geomorphometric data

We compiled total phosphorus (TP; mg L^−1^), total nitrogen (TN; mg L^−1^), dissolved organic carbon (DOC; mg L^−1^), and dissolved oxygen (DO; mg L^−1^) measurements from sampling observations which also presented, at a minimum, lake chla data, sampling date and geographic coordinates (Tables 2; 4). The methodology used to obtain *in situ* water chemistry data varied among studies and is described in the *methodsData.csv*. Generally, water chemistry samples were analyzed spectrophotometrically, fluorometrically, or by a multi-parameter water quality probe (e.g. Yellow Springs Instrument, which was supplemented with additional calibration methods to ensure measurement accuracy). We also collected lake volume, surface area, mean depth, maximum depth, Secchi depth, and pH from the original data provider when available within the study (Tables 2; 4). Secchi depth was often measured using either a 30 cm white circular disk or a slightly modified 20 cm disk with black and white patterning. Almost all studies used portable pH meters to measure water pH.

**Table 4:** Means and ranges of lake characteristics and water chemistry.

Variable	Units	Mean	Range	Sample size (n)
Year	—	2002	1933–2019	228,168
TN	mg L^−1^	0.908	0–20.6	39,457
TP	mg L^−1^	0.042	0–3.6	111,872
DO	mg L^−1^	9.82	1.32–67.7	761
DOC	mg L^−1^	0.008	0.01–1	10,517
Max depth	meters	15.6	0–310	188,205
Mean depth	meters	7.00	0.2–154	72,786
pH	—	7.99	5.5–10.7	12,934
Secchi depth	meters	2.76	0–61.7	195,782
Surface area	squared kilometers	25.11	<0.001–32,056	211,975
Chla	mg L^−1^	0.017	0–4.33	228,168

### Unique identification

We assigned a unique identifier (hereafter survey instance, labeled “uniqueID”) in the dataset to every chla data point separated by unique lake, GPS coordinate, year, month, and study. We could not treat every spatial coordinate as an independent lake because some coordinates were surveyed within the same lake either within or among studies. To determine unique lake identifiers that correspond with each survey instance, we used the HydroLAKES database of lake location and shape^30^ (http://wp.geog.mcgill.ca/hydrolab/hydrolakes/). We matched the spatial polygons of lakes present within the HydroLAKES database with the spatial coordinates extracted from the studies. In instances where the survey instance did not match a lake within HydroLAKES database, we conducted a Google search to determine if the lake was unique from others. Using these methods, we generated a unique lake identifier associated with each of our survey instances. The country was determined from the geographic coordinates of the lake.

## Data Records

We have published the MS_citations and Repo_citations in an open access repository^31^ (Filazzola *et al*. 2020. Knowledge Network for Biocomplexity. 10.5063/F1RV0M1S) with data from the published manuscripts and data repositories (Table 1) that were systematically processed to extract chla data (Table 3). Each of these files contains citation information such as the authors, year that the study was published, location published (e.g. journal, data repository), and whether the dataset was ultimately included within this database (Table 3). Each of these files lists studies that were explored as potentially having chla data but were excluded.

The main dataset file ChlaData.csv contains general information about each survey instance that connects across the other files by the uniqueID identifier (Table 3). The first column has a unique identifier that corresponds with every survey instance that is separated by year, month, geospatial point, and study. This file is to be used for subsetting the survey points for respective analyses, such as within a certain timeframe or country. This file also contains a column of lake identifiers corresponding to each of the survey instances because, within and among studies, some lakes were surveyed multiple times at different locations. All water chemistry variables reported, including chla are reported in mg L^−1^ (Table 4). Finally, the dataset includes information about the morphometric lake characteristics when reported, such as surface area, mean lake depth, and maximum lake depth (Table 4).

## Technical Validation

We conducted quality control and quality assurance across the database to validate the data from each of the independent sources. In total, there were 228,168 unique survey instances that required quality assurance and quality control (QA/QC). We separated our QA/QC into three distinct stages: 1) Import and Compilation, 2) Unique Lake Identification, and 3) Value Validation and Conversion.

### Import and compilation

Data extraction from each study was conducted by separate individuals and contributed to a master data file. After the data were assembled from each of the manuscripts and online repositories, we conducted initial examination of transcription errors such as variables placed in the wrong columns, variables missing units, or incorrect characters. To ensure there were no duplicates in data across the multiple datasets we examined samples collected in the same year, that were within 1 km of each other, and had chla values within 0.00005 mg L^−1^ of each other. A random subset of 10% from our database was validated by members within our group that were different than the original person that collected the data. These spot checks were meant to mitigate errors that could have been generated when compiling the database or converting values to the same units.

### Unique lake identification

We checked the coordinates for each survey instance to ensure it represented an actual body of water not in the ocean. When possible, the name of the lake described in the paper was compared to the described coordinates. Any errors or erroneous observations in location (e.g. negative longitudes for studies in the eastern hemisphere) were determined by comparing study descriptions with points and using a map of the lakes (Fig. 2). We compared maps of lakes within 1 km of each other but with different lake identifiers to ensure these were indeed separate lakes. To determine if any lakes were incorrectly identified as the same, we searched for any uniqueIDs that had the same lake identifier but were more than 100 km apart. In total, there were 1,374 lakes that we identified belonging to multiple studies such as Lake Taihu (20 studies), Lake Ontario (16 studies), and Lake Chao (8 studies).

### Value validation

We compared the distribution of all values to identify potential outliers that could indicate an incorrect measurement. The units across all datasets were standardized to all be mg L^−1^, and were converted from multiple other units including µg L^−1^, mg m^−3^, and g m^−3^. All lakes that had units mg m^−2^ were removed because they were based on downscaling of surface water only and did not convert properly to mg L^−1^. We rounded all values of chla to 0.0001 mg L^−1^ (0.1 µg L^−1^) because analytical equipment used within studies rarely had better precision. A full list of the sensitivities from each dataset can be found within the methodsData.csv dataset.

**Fig. 3:**
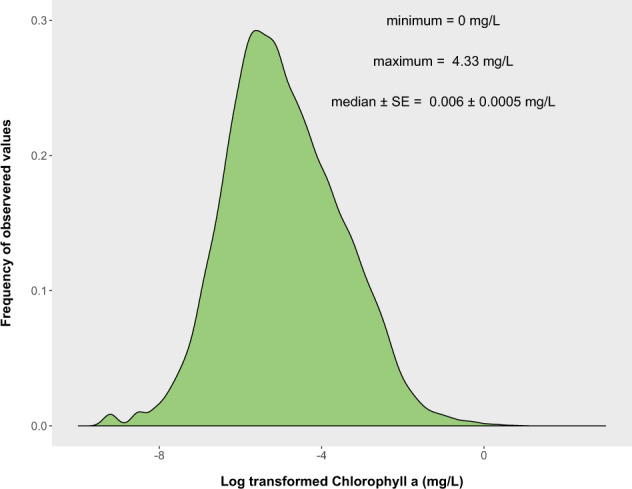
Frequency of observed chlorophyll values found in the lake dataset (n = 228,168).

**Fig. 4:**
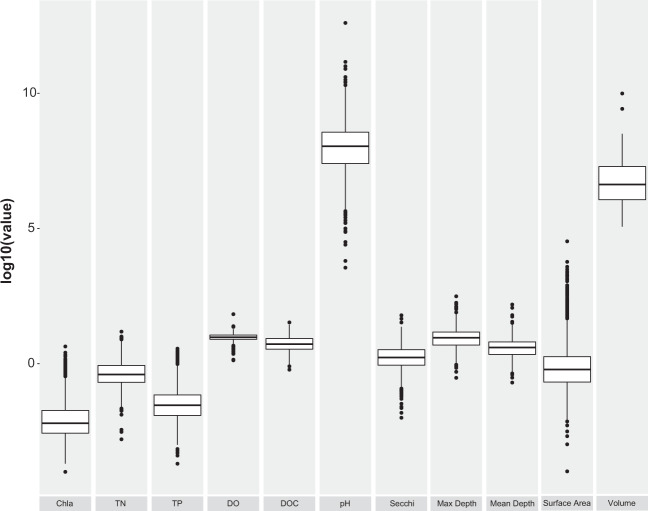
Distribution of water chemistry and lake morphometry values from database. Values represent log-transformed equivalent of the units presented in Table 4, except pH which is already log-transformed.

We generated histograms and compared the distributions of each variable to identify observations that could be erroneous. For all water chemistry variables, we flagged any observation that was above three standard deviations from the mean. These values were then compared to the original data source to ensure it was correctly transcribed. We conducted these flagging exercises excluding repo14 because that specific repository was extremely large relative to the other observations, is biased towards north-east USA, and has been extensively validated previously^25^. We explored all values that exceeded (>1 mg L^−1^) for the water chemistry variables. Many of the extremely high values (>1 mg L^−1^) were from a study by Marselina and Burhanudin^32^ that measured the water quality of extremely polluted lakes in Indonesia. The highest value recorded for chla was 4.33 mg L^−1^ taken from Binder Lake, Iowa in 2006 during what we believe was an algal bloom. We explored a log-transformed distribution of chla values and found the median chla value across all observations was 6.0 µg L^−1^ (Fig. 3). Approximately 30% of the observations were considered oligotrophic with chla values less than 2.5 µg L^−1^ (Fig. 3). Lakes with chla values of zero were observed in some arctic and alpine lakes. We observed two noticeable differences in symmetry in the histogram of chla that could potentially be explained by the detection limits of some devices for chla (e.g. 1.0 µg L^−1^) or trophic boundaries (e.g. oligotrophic vs mesotrophic). We also explored the distribution of the other water chemistry and lake morphometry variables (Fig. 4). Using boxplots, we examined outliers that may have not been flagged from comparisons of extreme values. By comparing observations outside of the boxplots, we identified values that may have suffered from conversion errors as these would typically be off by three orders of magnitude (e.g. 1 mg L^−1^ = 1000 µg L^−1^). Boxplots also allowed exploration of the distribution, to determine if there is any skew in the data that could have been generated by incorrect units or compilation errors. Any observations that were flagged were checked by exploring the initial dataset from which the values were obtained.

## Usage Notes

We provide code in R Version 3.5.1^33^ within our guide to join all files by their unique identifier for further analysis. Data synthesis and technical validation was conducted using tidyr^34^, and dplyr^35^. Visual quality assurance and figures were generated using ggplot2^36^. To compensate for some missing lake characteristics that were not reported in the searched manuscripts, such as lake volume, depth, or surface area, we suggest that authors use additional resources such as LakeNet (http://www.worldlakes.org/), Global Water Bodies database – GLOWABO^37^, International Lake Environment Committee Foundation – ILEC (http://www.ilec.or.jp/en/), Global Lakes & Reservoirs Repository – GLR (http://www.worldlake-db.com), NHDPlus Version 2 (https://www.horizon-systems.com/NHDPlus) or HydroLAKES (http://wp.geog.mcgill.ca/hydrolab/hydrolakes). The HydroLAKES database is particularly useful and provides additional geomorphic data for approximately 1.4 million lakes globally^30^ (https://www.hydrosheds.org/). For other lake characteristics, the Global Lake Area, Climate, and Population (GLCP) dataset has synthesized climate and human population densities for more than 1.4 million lakes globally^4^. These datasets can complement the chlorophyll database built here to explore factors that drive water quality in freshwater lakes.

## Data Availability

All code for analyses included within this manuscript as well as meta-data files (including unique identifiers, repository and manuscript data, lake characteristics, water chla and chemistry data, and water sample collection method) are provided in an open access repository^38^. Within the repository, we also provide code for unit conversion (e.g. µg L^−1^ to mg L^−1^), and extracting climate data from the Climatic Research Unit at the University of East Anglia (http://www.cru.uea.ac.uk/).
